# Using meta-quality to assess the utility of volunteered geographic information for science

**DOI:** 10.1186/s12942-017-0113-9

**Published:** 2017-11-06

**Authors:** Shaun A. Langley, Joseph P. Messina, Nathan Moore

**Affiliations:** 1 Urban GIS, 1143 W Rundell Pl Suite 301, Chicago, IL USA; 2 Center for Global Change and Department of Geography, Environment, and Spatial Sciences, Michigan State University, East Lansing, MI USA

## Abstract

**Background:**

Volunteered geographic information (VGI) has strong potential to be increasingly valuable to scientists in collaboration with non-scientists. The abundance of mobile phones and other wireless forms of communication open up significant opportunities for the public to get involved in scientific research. As these devices and activities become more abundant, questions of uncertainty and error in volunteer data are emerging as critical components for using volunteer-sourced spatial data.

**Methods:**

Here we present a methodology for using VGI and assessing its sensitivity to three types of error. More specifically, this study evaluates the reliability of data from volunteers based on their historical patterns. The specific context is a case study in surveillance of tsetse flies, a health concern for being the primary vector of African Trypanosomiasis.

**Results:**

Reliability, as measured by a reputation score, determines the threshold for accepting the volunteered data for inclusion in a tsetse presence/absence model. Higher reputation scores are successful in identifying areas of higher modeled tsetse prevalence. A dynamic threshold is needed but the quality of VGI will improve as more data are collected and the errors in identifying reliable participants will decrease.

**Conclusions:**

This system allows for two-way communication between researchers and the public, and a way to evaluate the reliability of VGI. Boosting the public’s ability to participate in such work can improve disease surveillance and promote citizen science. In the absence of active surveillance, VGI can provide valuable spatial information given that the data are reliable.

## Background

We are standing on the apex of a scientific transition as technological and communications barriers are toppled [1, 2], and the distinction between amateur and professional scientist is eroded. Neogeography characterizes the “blurring of the distinctions between producer, communicator, and consumer of geographic information”; the separation of scientist and layperson, expert and novice, is obscured as citizens engage in the generation of new knowledge [3]. As citizens engage in Science, we need to reconsider our traditional notions of authority, expertise, and purpose.

Neogeography, a type of citizen science, is the democratization of geographic tools and methods for non-traditional mapmaking. It has garnered a great deal of attention in the literature as we struggle to conceptualize the nature of “geographic expertise”; however, the involvement of citizens in science has long been established [3, 4]. Participatory science has sought to involve citizens directly in academic research and related exploits [5–7] on the premise that citizens are more informed actors with respect to their local environment than researchers operating externally. Citizens are perceived to hold authority through experience and status, and are acknowledged for their capacity to convey unique understanding, or indigenous knowledge [1, 5].

With the advent of Web 2.0 [8, 9] and the widespread availability of new technologies [6, 10], citizens are increasingly exposed to geographical information. Citizens also increasingly volunteer spatially explicit (geographical) information that is of relevance or interest to them, often integrating this information with existing datasets, or mashups, utilizing it for their own gain [4, 11]. Boulos [12, 13] first introduced this concept of collaboratively developed spatial information as the “Wikification of GIS by the masses”. Goodchild coined the term “volunteered geographic information” (VGI) to refer to spatial data that is contributed by ordinary citizens, irrespective of their training in scientific methods [14]. The notion of VGI grew out of recognition of the limitations of traditional methodologies for adequately mapping and assembling spatial information around the world that provided both good coverage and fine temporal resolution [15–17]. As a framework, VGI encompasses citizen participation from a range of social classes and computing practices with the express purpose of harnessing the collective intelligence [5, 18]; it builds on the notion that data can be shaped by social and political processes and an individual’s expertise, context, and spatial awareness [15, 19–21]. Local knowledge is crucial to an accurate geographic description of communities and social groups, involving the citizen in the process of data collection.

VGI in practice is now commonplace, e.g. Google Maps. Arguably one of the most successful, if not the most widely cited, outlet for VGI has been Wikimapia [14, 16]. Here individuals contribute knowledge of the physical, built environment around them in order to create as accurate a representation as possible. Recent events have also demonstrated the potential for VGI to assist in disaster response [22].

However, the utility of VGI remains limited. In the context of the broader GIS literature, data quality has always been a concern [16, 23]. In the case of VGI, this concern is exacerbated due to the lack of expertise, or credibility, of the individual [23]. Given that VGI is user-generated information by non-experts, there is no quality assurance of the data [24]. Others have raised concerns over the motivations of the individual, whether data is volunteered with intent to inform or mislead, an act of digital vandalism [25].

Many approaches have been taken to assess the quality and reliability of VGI [e.g. 10, 20, 23, 26], but mainly conceptual. The most common of these methods involves social trust networks and reputation models [10, 27]. Under this approach, data quality is checked by other project participants for errors and inconsistencies. In this model, no single expert is tasked with reviewing each volunteered report. Another approach recommended has been to use existing data sets (collected using more authoritative methods) to check for inconsistencies in data. However, quality is not absolute; a datasets fitness-for-use is contextual and may have varying degrees of suitability for different users [28]. No single metric can be used to determine whether a data set is suitable across all ranges of potential uses. Thus, the context of a user’s participation and interaction with VGI must be taken into account when considering accuracy/quality of VGI.

Given the concerns raised over the uncertainty of data quality in VGI, there is significant debate as to the utility of VGI for science. Elwood et al. [16] inventoried 99 projects utilizing VGI and found only 3% to have academic affiliations. One of the most prominent examples of VGI in science is the Audubon Society’s Christmas Bird Count. This project has amassed a significant volume of volunteered data; however despite attempts to train volunteers in data collection, lingering questions of data quality, of reliability, have limited any analytical value and integration potential with authoritative datasets [29].

The credibility (or believability) of VGI can be described objectively by traditional measures of data quality—the degree to which the information can be considered accurate, or as the subjective perception on the part of the consumer [23]. However, for VGI to be useful for science, it is the traditional, objective “credibility-as-accuracy” measure demanded [23]. To fully quantify error in data, it is necessary to have a measure or to make assumptions as to the nature of the population being measured, to compare the distribution of data against the population as a whole. It is in this way we measure attribute accuracy, completeness, thematic resolution, and variability, to name only a few. Other measurements rely on feedback from measurement equipment, such as positional accuracy, temporal accuracy, spatial and temporal resolution, among others. Participatory science and VGI Science (VGIS) often involve datasets for which the nature of the population is not immediately known. Therefore, a direct quantification of the error of VGI is only possible in a post hoc analysis. However, it is the immediate benefit VGI can provide us that is of interest here and so we must develop a mechanism to evaluate the merits of VGI in real time (as it is contributed). In the absence of an ability to directly measure error and uncertainty parameters of volunteered data, we can use a surrogate measure, *meta*-*quality*, a measurement of the collective quality of the data [30].

The objective of our work here is to improve the perceived value of VGI for science by demonstrating a methodology for VGI data quality assessment. We accomplish this through a mechanism to explicitly assess the reliability of reporters based upon their respective VGI contributions.

To better illustrate our approach, we apply the methodology to a case study in disease ecology where we model the distribution of the tsetse fly, the principle vector of African Trypanosomiasis in sub-Saharan Africa. The “Tsetse Ecological Distribution model” or TED is based on an assessment of environmental characteristics critical for the persistence of the fly [31]. The model is a conservative estimation of the population distribution specifically minimizing errors of commission; therefore, the TED model is an estimation of the minimum extent of tsetse at each point in time. However, the model is reliant on a static land cover classification and makes no adjustment for error intrinsic to the model [31]. The TED model produces estimates of the spatial distribution as binary outputs indicating presence/absence of the fly for each time period.

Potentially the most important contribution to incorporating VGI into a species distribution model of the kind here is the fact that we can explicitly address one component of model error (omission) without contributing additional error. TED was developed as a conservative model of the minimum expected distribution of tsetse. By incorporating VGI into the model results, we can effectively facilitate the population expanding over gaps of unsuitable habitat, either due to actual conditions or poor input data. It is known that microclimates provide refuge for tsetse in areas where the habitat would be otherwise unsuitable [32, 33]. The spatial resolution of the underlying MODIS data misses these microsites and therefore omits these cells in the estimated distribution. Allowing the distribution to be updated based on the VGI would allow us to more accurately reflect conditions as they exist reflecting sub-pixel dynamic that otherwise would not be possible. Incorporating VGI into the model results to expand the distribution can therefore reduce errors of omission without contributing additionally to errors of commission, thereby reducing total error, and thus improving data quality. Incorporating VGI into TED requires two distinct steps: (1) determine the reliability of the reporter to assess whether the VGI meets the threshold for acceptance, and (2) update the tsetse distributions by changing the binary tsetse presence/absence value for the cell (in which the datum is located) to 1—indicating presence of the fly. In cases where VGI reflects the predicted distribution, no change is made.

## Methods

Here we undertake a series of experiments to illustrate the integration of VGI into a traditional analytical model. First, we explore the characteristics of VGI and its impact on model results. Second, we evaluate the sensitivity of the model to three types of error common to crowdsourced data. Finally, we explore the importance of reliability, as measured by a reputation score [26, 27, 34] in determining the threshold for accepting the data for inclusion in the model, under both static (a pre-defined score) or dynamic (a varying score) conditions.

To simulate the generation of VGI, we first consider the different kinds of reporters and the characteristics of the data they might contribute (Table 1). We identify four basic types of reporters: (1) “always right”, (2) “always, intentionally wrong”, (3) “random”, and (4) “normal”. The “always right” reporter represents individuals who are judged, post hoc, to be highly reliable and the data they contribute are of high quality, often promoted to the role of moderator in online forums [27]; there is no (or minimal) spatial or temporal error component to the data they contribute. The “always, intentionally wrong” reporter represents individuals who consistently, and/or intentionally provide erroneous data [35, 36]; these reporters are unreliable and the data they contribute should always be rejected. The “random” reporter represents individuals who generate data, falling on a random distribution, reporting tsetse fly presence, for example, at apparently random locations across the landscape (whether or not they are actually present) ignorant of underlying habitat conditions [37, 38]; due to the random nature of the reports, the data are therefore unreliable. Finally, the “normal” reporter represents the typical individual who volunteers information; the individuals have a high degree of credibility and the data are usually high quality [23], but there is a spatial and temporal error component to the data they contribute. It is this type of reporter that we are most interested in evaluating reliability.

**Table 1:** Reporter types and the criteria used to simulate their behavior

Reporter	Type	Model criteria
1	Always right	Tsetse predicted
2	Always, intentionally wrong	Tsetse not predicted, habitat unsuitable
3	Random	Spatially random
4	Normal	Suitable habitat + one occupied neighbor

In the context of our case study, the simulated data for each reporter are based on habitat suitability criteria. In a real scenario, it is not possible to assess the accuracy of any report by itself; rather we can only assess the fitness-for-use of the data by placing it in application context and asking whether it is plausible [39, 40]. We simulate this by evaluating the data based on the likelihood of the data being correct given the underlying habitat conditions. To simulate the data, we identify a set of conditions that would be consistent with reports made for each reporter type, and use these conditions to identify points that can be used in our sample data set. Table 1 fully describes the types of reporters and the set of conditions used to simulate data. For completeness, we explore the impact on the predicted occurrence of tsetse by simulating data, not only from the four reporter types but also from data generated from all combinations of habitat suitability criteria. It is based, in part, on these simulations that we identified the specific combination of criteria that would be used to render simulated VGI (Table 2).

**Table 2:** Simulation results for simulated conditions

Sim	Criteria	% Gain				Variance			
		Overall	2004	2005	2006	Overall	2004	2005	2005
1	Random	9.81	4.23	13.66	11.85	144.02	76.83	105.73	106.62
2	Suitable habitat	14.06	7.22	17.94	17.58	108.26	73.41	80.88	77.11
3	One neighbor	0.29	0.17	0.39	0.32	93.37	27.62	66.28	65.48
4	Suitable habitat + one neighbor	0.03	0.02	0.04	0.05	19.65	10.86	16.52	13.97
5	Tsetse present	0	0	0	0	0.01	0.01	0.01	0.01
6	Tsetse not present	10.59	4.78	14.57	12.71	128.16	75.51	97.03	91.45
7	Habitat unsuitable	8.23	3.46	11.75	9.66	138.43	79.15	112.02	109.58

Values represent percent increase over the base TED model

The simulated data are based on the underlying conditions present at each time step in the model, but not necessarily on the predicted occurrence for that simulation. For each set of criteria and combination thereof, we ran 100 simulations, identifying 100 points in each time step to serve as mock reports. Pooling these data points together results in 10,000 potential locations (some locations are represented more than once in the pool due to random selection in the simulations) for reports for each time step from which we randomly draw from when simulating reporters. This allows us to incorporate a minimum amount of stochasticity that would exist with reporters in a real-world scenario.

The basic TED model was implemented in GRASS based on the methods outlined by DeVisser et al. [31]. Building on our implementation of the TED model, we model the predicted distribution of tsetse, incorporating VGI, and evaluate the magnitude of the difference. Each model was written in BASH, a UNIX shell-scripting language. The models were run on the High Performance Computing Center (HPCC) cluster at Michigan State University for a total of 9321 simulations representing an estimated 13,981 h of computing time.

The normal reporter is defined as an individual who usually provides credible data, but has the potential to submit erroneous data. Incorporating these inaccuracies into the data stream produces some degree of error in the model output. In reality, it is not possible determine the truthfulness of the data; therefore we must be able to determine the influence of error on the model output. The standard “normal” reporter is assigned an error rate of 10% (an arbitrary assignment); we measure the effects of this error by evaluating the impact on the resulting distribution when the “normal” reporter is assigned an error rate of 50%. The arbitrary choice would likely have an impact on the results because higher error rates would require more trials to identify credible reporters. However, since this presents a proof-of-concept just to see if the process works, we did not perform a sensitivity analysis on these error rates yet. As the data are constructed based on the combination of habitat suitability criteria, we evaluate introducing error into the model in different ways. Erroneous data are simulated by selecting points in areas of unsuitable habitat by shifting the location of the point (simulating positional error), or by holding the data until the following time step (simulating temporal error). A z-score is computed comparing each set of criteria against a simulation where points are selected at random, as well as a test of significance against the output from the TED model alone (no VGI data incorporated).

An assessment of the reliability of the VGI requires us to first generate a dynamic history for each reporter that reflects the plausibility of the data as determined by habitat suitability criteria. Each reporter is assigned a score, a measurement of their reputation, which is a product of these criteria (slightly modified from Langley and Messina 2013 [26] to allow for negative changes in reputation). The index returns an ordinal measurement of reliability; it is not constraint to a particular range, rather is structured such that positive scores convey reliability. It is computed as:

1 $$Reliability = \theta + \rho + \frac{\kappa }{4} + \gamma$$

*θ* = reporter’s score, *ρ* = the number of times a cell was previously occupied (− 1 if 0), *κ* = the number of occupied cells in 4-cell neighborhood (− 1 if 0), *γ* = the number of supporting reports (− 1 if 0).

We arbitrarily selected threshold scores of 5 and 8 for incorporation of the VGI into the TED model results. This arbitrary choice would affect results when exercised in a real-world case; however, for our purposes, we merely needed threshold scores of any value to see whether or not the process actually worked. Higher or lower threshold scores would just require fewer or more trials to assess correctness. A paired *t* test is used to measure the significance of adjusting the threshold and the potential importance the specific selection has on the resulting predicted occurrence. An alternative approach to the arbitrary assignment of scores is to determine the threshold at which reporter types can be distinguished from each other. We subject the history of reporter scores to a k-means test; this analysis tries to iteratively place each reporter into one of two clusters (we define these clusters to mean reporters of “plausible” or “erroneous” data). Cluster centers were defined at random from the set of scores for each test. As reporter scores increase over time, we expect it will take a certain number of model time steps before they will group properly. The average reporter score (for the plausible group) from 100 iterations can be interpreted as a reasonable threshold score under a static model.

Over time, the scores for reporters quickly exceed the small thresholds we set (reaching values > 100 at the end of the simulation), which results in unqualified acceptance of the VGI into the model. As such, we cannot detect or respond (within a reasonable time) to changing behavior among reporters, reflecting the inability of arbitrary, static thresholds to capture potential declining reliability and reputation of reporters over time. In the final set of simulations, we explore the possibility of using a dynamic score model, where the threshold for acceptance is drawn from the distribution of all reporter scores at each time step. For each simulation, we set a threshold equal to the 1st quartile score, mean, or 3rd quartile score from the distribution of all reporters’ scores at that time. This allows us to include only the most reliable reporters from our total pool of participants, and the longer the model operates over time, the more reliable our output becomes. The net benefit to the model should thus improve over time. Sets of paired t-tests are used to measure the significance of the difference in predictions from the three threshold models.

In our case, the likelihood that tsetse are present in an area (the subject of the VGI in question) is correlated with the habitat suitability as measured by land cover, land-surface temperature, and NDVI (Normalized Difference Vegetation Index). A reporter’s score is a measurement of their reputation, akin to eBay’s ratings system, which quantifies the history of the individual to perform in a manner that is perceived positively by their peers [27]. We assume that if a reliable reporter contributes information that confirms another’s data, the likelihood that datum being accurate is improved. However, this method of confirmation by peers necessitates a set of reporters who have attained a data history. Until a reporter attains a certain reputation, we do not have enough information to assess data quality; however, we have seen that different reporters themselves quickly separate from each other, allowing us to partition out individuals who are either reporting randomly (and thus frequently inaccurately) or are simply providing erroneous data intentionally. Partitioning out these two types of reporters alone immediately improves the quality of the contributed data.

## Results

Varying the criteria for spatially locating VGI greatly influences the overall impact on the predicted occurrence of tsetse, however the impact varies markedly from year to year due to environmental conditions and shifts in the habitat suitability. Randomly locating points results in an overall 9.81% (4.23–13.66% for individual model years) increase in the number of cells in which tsetse are predicted to occupy over the time period in the model (recall that incorporating VGI into the TED model can only increase the prevalence of tsetse). However targeting specific locations where habitat is suitable and at least one neighbor is predicted to be occupied (the criteria we assign to our normal reporter), yields an overall 0.03% (0.02–0.05%) increase in occupied cells. Notably, selecting suitable habitat alone as our criteria influenced the results the most, with an overall 14.06% (7.22–17.94%) increase in predicted occurrence. Likely this speaks to the design goal of the TED model to minimize errors of commission. Predictably, constraining report locations to only those cells in which tsetse are predicted to occur (the condition for our “always right” reporter) yields no increase in the predicted occurrence of tsetse over the base model. Selecting locations in which tsetse are not predicted to occur or where habitat is unsuitable (conditions for the “wrong” reporter or a component of error in the normal reporter, respectively) yields an overall 10.59% and 8.23% increase in the predicted occurrence. All criteria tested yielded significantly different results over the random model (*p* < 0.001 in each case).

In the static threshold score model, there was no significant difference in the overall predicted occurrence of tsetse (*p* > 0.4). However, utilizing a dynamic threshold score model resulted in significant differences between all three models (1st quartile, mean, and 3rd quartile) with *p* values < 0.001 in each case. The overall increase in predicted occurrence was 0.8, 0.43, and 0.12% respectively; however, the results varied widely from year to year for both static and dynamic threshold models (see Table 3). [Note: simulations 8 through 12 in the table consider the cases for only normal reporters].

**Table 3:** The percentage increase in the prevalence of tsetse over the base TED model for simulations 8–12

Sim	Score	% Gain				Variance			
		Overall	2004	2005	2006	Overall	2004	2005	2006
8	5	1.28	0.27	1.94	1.68	139.56	46.8	113.56	100.52
9	8	1.22	0.23	1.88	1.6	138.6	44.09	112.57	99.1
10	1st quartile	0.8	0.13	1.15	1.2	120.62	37.76	92.9	95.07
11	Mean	0.43	0.05	0.6	0.68	109.46	28.92	83.06	83.27
12	3rd quartile	0.12	0	0.14	0.24	77.36	10.47	55.49	66.11

**Fig. 1:**
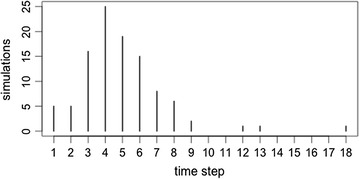
A frequency plot representing the time-step in which reporters cluster into two groups, for 100 replications of simulation 13

The four types of reporters cluster into two groups—see simulations 13 and 14 (Table 4) for the cases where all reporter types are considered. The four reporters are not fully distinguishable from each other at any time in our models (k-means with four clusters). Figure 1 presents the distribution curve (for all 100 replications) for the time step, at which point the reporters can be distinguished using a k-means clustering approach. For simulation 13, where a threshold score of 5 is used, the reporters can be separated, on average, in the 5th time step (mean = 4.93, median = 5). The average reputation score in the 5th time step is 10.87 for the “plausible” group. Reporters in simulation 14 (50% error rate) do not consistently cluster together into two groups.

**Table 4:** The percentage increase in the prevalence of tsetse over the base TED model for simulations 13–20

Sim	Error type	% Gain				Variance			
		Overall	2004	2005	2006	Overall	2004	2005	2006
13	10%	1.38	0.39	2.07	1.74	139.44	48.86	116.26	95.18
14	50%	5.23	1.88	7.94	5.95	144.9	70.59	124.32	99.84
15	Spatial shift 5%	1.39	0.39	2.13	1.7	137.33	49.43	112.58	92.83
16	Spatial shift 10%	1.39	0.44	2.22	1.52	142.05	54.59	118.36	97.97
17	Spatial shift 25%	1.41	0.44	2.18	1.65	131.66	50.61	108.22	95.31
18	Temporal shift 5%	1.46	0.43	2.21	1.79	135.71	50.68	112.63	97.89
19	Temporal shift 10%	1.54	0.45	2.4	1.81	149.88	52.54	124.28	97.73
20	Temporal shift 25%	1.61	0.5	2.47	1.9	148.95	55.65	119.68	97.55

The arbitrary 10% error threshold

**Fig. 2:**
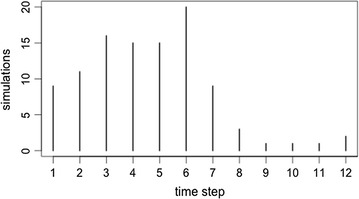
A frequency plot representing the time-step in which reporters cluster into two groups, for 100 replications of simulation 10

**Fig. 3:**
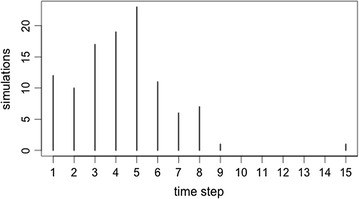
A frequency plot representing the time-step in which reporters cluster into two groups, for 100 replications of simulation 11

**Fig. 4:**
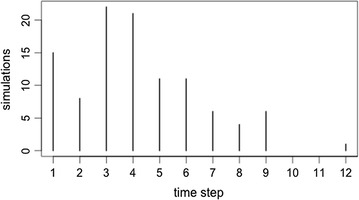
A frequency plot representing the time-step in which reporters cluster into two groups, for 100 replications of simulation 12

Considering the dynamic score models, there were no significant differences in the time needed for reporters to group together. For the 1st quartile threshold score (simulation 10), reporters clustered into two groups, on average, in the 5th time step (mean = 4.61, median = 5). The average score for the “correct” reporters in the 5th time step was 18.87 (Fig. 2). In the mean threshold score models (simulation 11), reporters clustered together in the 4th time step (mean = 4.32, median = 4). The average reputation score for reporters in this time step was 15.06 (Fig. 3). Finally, for the 3rd quartile threshold score model, reporters clustered together in the 4th time step (mean = 4.21, median = 4) with an average reputation of 15.14 (Fig. 4).

The nature of error (positional vs. temporal) introduced into our models through incorporating VGI did not appear to change the magnitude of the impact on predicted occurrence. This was also true when varying the magnitude of the error, at least for the range tested (5–25%). We did observe a significant increase in the predicted occurrence of tsetse when the magnitude of the error introduced was 50% (where each reporter had a 50% chance of contributing erroneous data); introducing error of any type, though, results in a significant increase in the predicted occurrence compared to the case where no error is considered (simulation 4). Therefore, at least in our case study, the error introduced from VGI is not expected to a statistically significant effect on the prevalence of tsetse. This suggests that our models are resilient to the introduction of some erroneous data. Adaptations of our model to different studies will nevertheless necessitate an exploration of the role of introduced error from VGI to assess the resiliency of scientific models.

**Fig. 5:**
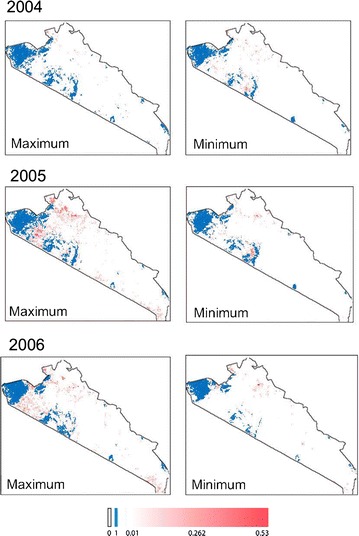
The theoretical maximum and minimum extent (respectively) for the distribution of tsetse for simulation 10. Values represent the proportion of time-steps in the model where tsetse were present; this is a rough approximation of the probability of tsetse occurrence

**Fig. 6:**
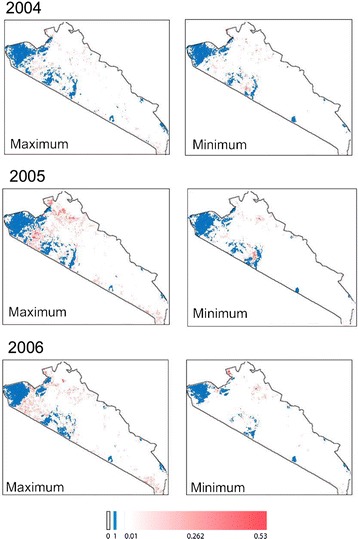
The theoretical maximum and minimum extent (respectively) for the distribution of tsetse for simulation 11. Values represent the proportion of time-steps in the model where tsetse were present; this is a rough approximation of the probability of tsetse occurrence

**Fig. 7:**
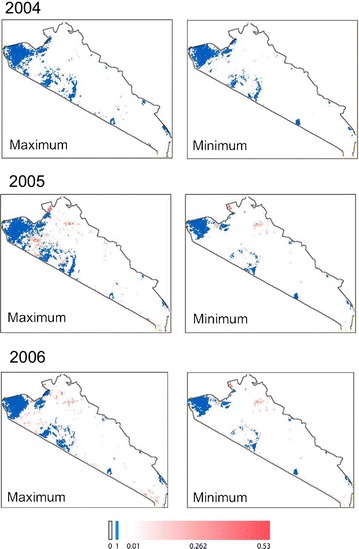
The theoretical maximum and minimum extent (respectively) for the distribution of tsetse for simulation 12. Values represent the proportion of time-steps in the model where tsetse were present; this is a rough approximation of the probability of tsetse occurrence

While the analysis reveals significant differences in the predicted tsetse occurrence from incorporating VGI into the TED model, global metrics are difficult to interpret given the importance of spatial structure in the dataset. To this extent, visualizing the structure of tsetse distribution patterns can lead to novel interpretations of the influence of VGI. Figures 5, 6 and 7 present the predicted distribution of tsetse over our study area (for simulations 10, 11, and 12 respectively); cell values indicate the proportion of time steps in the model (every 16 days between 2004 and 2006) where tsetse are predicted to occur, averaged across 100 replications. The distributions incorporating VGI closely mirror the base TED model with marked differences between core tsetse areas. These maps illustrate specific areas where VGI is particularly influential, likely due to the ability of tsetse populations to “jump” patches of unsuitable habitat.

Time is a significant factor to consider when evaluating the results of our models. In describing the output of TED model predictions, DeVisser et al. [31] noted that tsetse populations tended to reach their maximum extent at the end of the long rains (ending the beginning of June). Populations tended to reach their minimum extent at the end of the cool dry season (mid- to late-October). This interpretation of tsetse population distributions comports with what is observed in my simulations, and is grounded in an ecological understanding of tsetse population dynamics.

## Conclusions

Volunteered geographic information can make valuable contributions to science, enhancing datasets from more authoritative sources. However, integrating VGI data necessitates assessing the error and uncertainty of those data. Direct quantification of data quality in this context is difficult; the traditional components (e.g. accuracy, precision, and variance) typically cannot be ascertained for VGI. It is critical for us to at least be able to qualify data quality, as it serves as the foundation from which we assess fitness-for-use. We have proposed using reputation or reliability (of the reporter) as a surrogate measure of meta-quality. As an initial assessment, meta-quality allows us to begin to break through the cloud of uncertainty inherent with VGI.

**Fig. 8:**
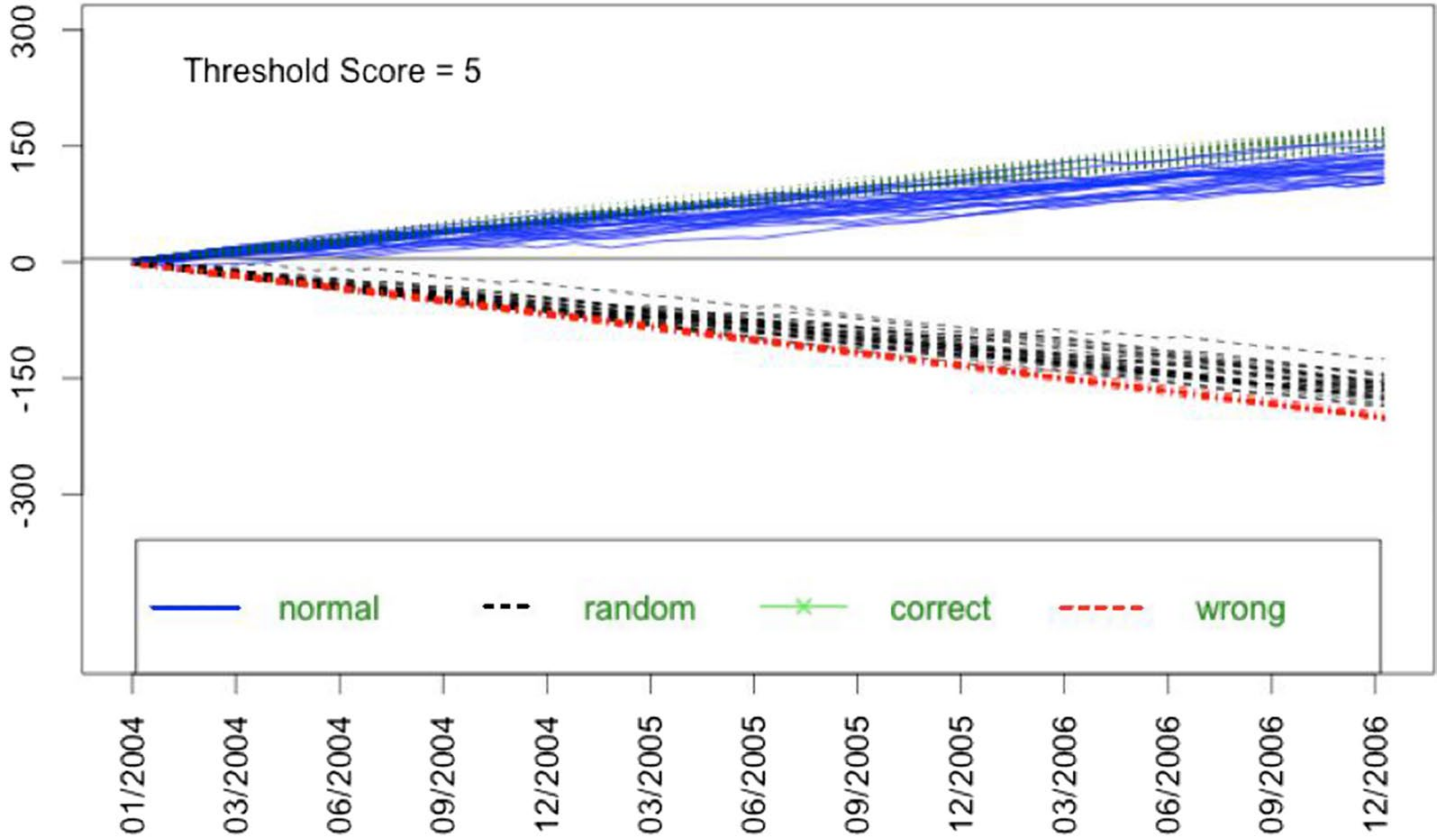
This figure overlays the scores of 100 reporters for simulation 8

We build on the power of the reliability/reputation assessment by considering a dynamic threshold-scoring model. While we considered three different criteria for establishing a threshold (defined as the 1st quartile, mean, and 3rd quartile values in the distribution of reporter scores in each time step), we did not find a significant difference between them—as measured by an overall increase in the prevalence of tsetse in our models. In considering only those individuals whose reliability exceeds the mean score for all reporters, we only incorporate VGI from a subset of reporters we deem the most reliable. As scores improve for all individuals (regardless whether we have incorporated their data into our models), the threshold for acceptance/inclusion in our models also increases (approximately linearly in our models—Fig. 8 shows the trend for one simulation). Over time, the quality of VGI data that we incorporate will improve, and the impact of any erroneous data we have included should decrease. Most importantly, a dynamic threshold model facilitates detection of declining performance (of a reporter) and a rapid response to limit the acceptance of poor quality data. Figure 8 illustrates that over time, random or erroneous reporters get consistently lower scores, with accurate reporters get consistently higher scores. This shows that this approach produces strong and clear divergence separating out erroneous reporting.

The potential value of a means to assess data quality of VGI is immense. The strongest hurdle to fully utilizing VGI has been our inability to measure data quality and uncertainty. In demonstrating a valuation system for VGI (based on the reputation of reporters themselves), we have, in part, overcome this hurdle. To date, the utilization of VGI for science has been reserved for those cases only where the performance of reporters is controlled through training and guidance while closely monitoring the entire process from data collection to communication [7, 20, 29]. But this runs contrary to many of the perceived strengths of VGI, the dissolution of traditional roles [1, 3, 6, 41] and the establishment of a two-way communication model for geographical information [14]. Projects that have tried to embrace VGI have done so under the old model of participatory science, and thus are subject to all the perceived and actual limitations [5, 11]. Many factors influencing quality remain difficult to measure, including rates of participation and motivation to participate; the value of VGI cannot be fully appreciated until we can reliably assess these factors and the role they play in determining data quality.

It is our position that incorporating VGI into standard scientific models, particularly those where available data are sparse, can significantly improve the performance of the models and the predictive or explanatory power of the results. Consider the case of “Digital Earth”; first conceived by then US Vice-President Al Gore, it represented a push to represent the planet in high-resolution, multi-dimensional space for the primary purpose of improving our predictive capabilities of Earth’s ecosystems [24, 42]. Twelve years later, significant gaps still exist, particularly in terms of our capacity to collect certain types of data of sufficient quality and resolution [42]. Harnessing the collective power of earth’s citizens, the aggregate power of “six billion sensors”, we can make significant strides to improving the predictive capacity of our models through incorporating new types of information [14]. Therefore, it is critical we continue to explore ways to assess the credibility of VGI, to embrace the new geographical traditions, while respecting the scientific paradigms of the past.
